# Altered Metabolic Networks in Mesial Temporal Lobe Epilepsy with Focal to Bilateral Seizures

**DOI:** 10.3390/brainsci13091239

**Published:** 2023-08-25

**Authors:** Zhihao Guo, Jiajie Mo, Jianguo Zhang, Wenhan Hu, Chao Zhang, Xiu Wang, Baotian Zhao, Kai Zhang

**Affiliations:** 1 Department of Neurosurgery, Beijing Tiantan Hospital, Capital Medical University, Beijing 100070, China; guozhihao1995@foxmail.com (Z.G.); jiajiemo@foxmail.com (J.M.); zjguo73@126.com (J.Z.); huwenhan88@163.com (W.H.); babaoriley1@163.com (C.Z.); wang19001@163.com (X.W.); zhaobaotian0220@foxmail.com (B.Z.); 2 Department of Functional Neurosurgery, Beijing Neurosurgical Institute, Capital Medical University, Beijing 100070, China

**Keywords:** mesial temporal lobe epilepsy, focal to bilateral tonic-clonic seizures, metabolic network

## Abstract

This study was designed to identify whether the metabolic network changes in mesial temporal lobe epilepsy (MTLE) patients with focal to bilateral tonic-clonic seizures (FBTCS) differ from changes in patients without FBTCS. This retrospective analysis enrolled 30 healthy controls and 54 total MTLE patients, of whom 27 had FBTCS. Fluorodeoxyglucose positron emission tomography (FDG-PET) data and graph theoretical analyses were used to examine metabolic connectivity. The differences in metabolic networks between the three groups were compared. Significant changes in both local and global network topology were evident in FBTCS+ patients as compared to healthy controls, with a lower assortative coefficient and altered betweenness centrality in 15 brain regions. While global network measures did not differ significantly when comparing FBTCS− patients to healthy controls, alterations in betweenness centrality were evident in 13 brain regions. Significantly altered betweenness centrality was also observed in four brain regions when comparing patients with and without FBTCS. The study revealed greater metabolic network abnormalities in MTLE patients with FBTCS as compared to FBTCS− patients, indicating the existence of distinct epileptogenic networks. These findings can provide insight into the pathophysiological basis of FBTCS.

## 1. Introduction

Temporal lobe epilepsy (TLE) is among the most prevalent subtypes of focal epilepsy among adults, and it often fails to respond to antiepileptic drugs, particularly in individuals exhibiting hippocampal sclerosis (HS) [1]. Focal to bilateral tonic-clonic seizures (FBTCS) affect an estimated one in three TLE patients [2], representing the most severe type of epileptic seizure and increasing the odds of seizure-associated injury or sudden unexpected death in epilepsy (SUDEP) [3]. FBTCS incidence is also associated with a poor prognosis for seizure freedom after the surgical treatment of epilepsy in individuals diagnosed with TLE. The mechanistic basis for FBTCS has yet to be established, particularly with respect to how this form of seizure only impacts a subset of TLE patients [4]. Thus, elucidation of the underlying mechanisms of FBTCS is essential and has the potential to improve future treatment strategies for TLE.

Many research efforts have focused on defining FBTCS-related risk factors, with HS having been shown to be positively correlated with this seizure type, whereas both pedal automatism and ictal speech are negatively correlated with FBTCS incidence [2,5]. The thalamus has been found to play a role in the pathogenesis of idiopathic generalized epilepsy (IGE), with IGE patients exhibiting a reduction in associated gray matter volume [6]. IGE patients with enhanced thalamocortical connectivity also present with structural anomalies [7,8]. FBTCS has been tied to damage to multiple areas of the brain, including the thalamus and basal ganglia circuits, ultimately contributing to both anatomical and functional abnormalities [9,10,11]. FBTCS has been proposed to exhibit a distinct etiological basis from that of IGE, with a greater degree of patient-specific spread [12]. Both structural and functional changes in the brain are thought to underlie the large spread of epileptic activity associated with this form of seizure. Efforts to fully elucidate these complex brain networks that extend beyond the typical thalamocortical circuits are thus vital to fully understanding the drivers of FBTCS [10].

A growing body of research indicates that even instances of so-called “focal” epilepsy involve large epileptic networks within the brain rather than truly being restricted to a particular focal area [13]. Mesial TLE (MTLE) has thus been posited to represent a network disorder involving many other regions of the brain outside the mesial temporal lobe [14,15]. TLE-related brain networks have been detected via functional and structural analyses, particularly in individuals with MTLE [16,17].

Graph-theoretic analytical strategies can be employed to define the topological organization of these connections [18], providing network-based insights into the pathophysiology of this condition. One such network measure is “betweenness centrality,” which is a fundamental concept in network analysis. Betweenness centrality quantifies the extent to which a specific brain region acts as a bridge for information flow between other regions in the network.

Functional connectivity can be analyzed through a range of strategies, such as functional MRI (fMRI), electroencephalography (EEG), magnetic encephalography (MEG), and fluorodeoxyglucose positron emission tomography (FDG-PET), while structural connectivity is primarily assessed via diffusion tensor imaging (DTI). In fMRI-based analyses, shifts in blood oxygenation and the regional flow and volume of cerebral blood are reflected by the BOLD signal, with these hemodynamic parameters being linked to neuronal activity through neurovascular coupling. FDG-PET-based analyses can detect energy consumption in the brain as a readout for neuronal communication, with these readouts being closely tied to functional connectivity [19]. These FDG-PETs can also provide lower levels of variance, a higher signal-to-noise ratio, and better replicability as compared to fMRI [20]. Several articles have employed MEG, fMRI, EEG, and DTI to assess functional and structural connectivity in focal epilepsy patients, whereas FDG-PET-based investigations focused on metabolic connectivity in TLE patients have been less common [21,22]. Graph theory has recently been leveraged to conduct a growing number of network analyses in MTLE patients with FBTCS through resting-state fMRI, task-based fMRI, and DTI approaches, with all of these strategies revealing altered network status [9,23,24]. To date, no analyses have specifically focused on using FDG-PET results to compare differences in the brain metabolic networks of MTLE patients with and without FBTCS.

The present study was designed to investigate the changes in the metabolic network in MTLE patients with and without FBTCS compared with healthy controls. Previous research has suggested that subcortical structures may play an intermediate role in secondary generalization. However, there is still insufficient research on the overall brain metabolic network differences between MTLE patients with and without FBTCS. Considering this, the study suggests that alterations in the metabolic connectivity of cortical and subcortical structures may be related to FBTCS. By delving into these alterations, it may be possible to gain a better understanding of the mechanisms underlying FBTCS in MTLE patients.

## 2. Materials and Methods

### 2.1. Participants

In total, this study enrolled 54 individuals diagnosed with unilateral drug-resistant MTLE who had undergone presurgical assessment at the Beijing Tiantan-Fengtai Epilepsy Center from 2016–2019. Comprehensive preoperative analyses of all patients consisted of the collection of a complete medical history, scalp EEG, neurological examinations, FDG-PET, high-resolution MRI scanning specific for individuals with epilepsy, and image postprocessing. In 24 patients in whom the extent and location of the epileptic zone could not be established during the initial evaluation, stereoelectroencephalography (SEEG) electrode implantation was performed. After evaluation, the epileptic focus was confirmed and lateralized to the unilateral hippocampus. Only those patients who had received a pathological diagnosis of HS were included in this study, while patients with focal cortical dysplasia, tumors, vascular lesions, or other pathological conditions were excluded. Seizure types included in the present study included both FBTCS and focal impaired awareness seizures (FIAS) [25]. Clinical FBTCS diagnoses were made based on a combination of historical data, EEG, and video-EEG monitoring. Individuals enrolled in the FBTCS− group were patients who only had a history of FIAS, while patients with a history of both FBTCS and FIAS were enrolled in the FBTCS+ group.

As a control group, 30 healthy individuals exhibiting normal neurological examination findings without any history of neurological, medical, or mental health issues were recruited.

The Ethics Committee of the Beijing Tiantan Hospital approved this study (KY 2020-052-02), and all participants provided written informed consent. 

### 2.2. ^18^F-FDG-PET Scanning

Patient PET scanning was performed in the interictal state, with identical scanning protocols for control individuals and MTLE patients. All ^18^FDG-PET scans were conducted with a GE Discovery ST PET-CT system using routine testing procedures (300 mm field of view, matrix 192 × 192, 3.27 mm slice thickness). Briefly, ^18^F-FDG was intravenously administered (average dose: 310 MBq/70 kg), after which patients were directed to rest for 40 min in a dimly lit room. The ordered subset expectation maximization algorithm was used when reconstructing PET images, with transmission scans from a germanium source being used to correct for attenuation. All PET scans were conducted within 6 months of patients undergoing epilepsy surgery evaluation and after patients had been seizure-free for 6 h, with no seizures occurring during scanning. 

### 2.3. Image Preprocessing and Graph Theoretical Analyses

**Figure 1:**
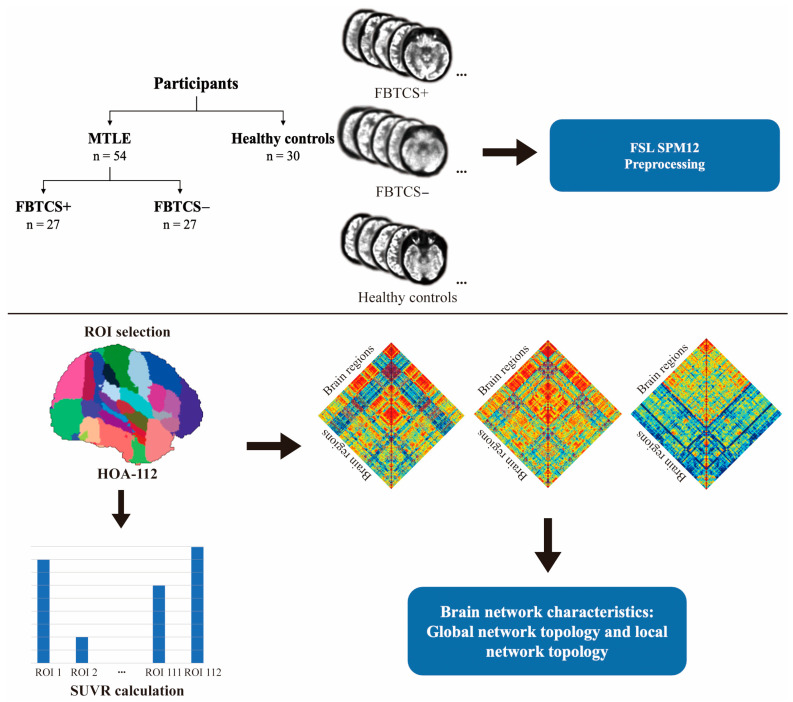
The FDG-PET-based metabolic connectivity analysis workflow. MTLE: mesial temporal lobe epilepsy; FBTCS: focal to bilateral tonic-clonic seizures.

An overview of the image preprocessing and graph theoretical analysis for this study is shown in Figure 1. PET image realignment and normalization were conducted with the FSL software package (FMRIB Software Library, Oxford University, Oxford, UK) and SPM12 (Wellcome Department of Cognitive Neurology, University College, London, UK). Steps involved in the preprocessing of PET images included skull stripping, matrix resizing, realignment, and normalization. SPM12 was then used for the spatial normalization of individual PET scans into the MNI space. Furthermore, the images of left-MTLE patients were reversed to make sure that the epileptogenic focus of all patients was on the right side. The right brain was treated as being ipsilateral to the epileptogenic focus, while the left brain was treated as being contralateral to the epileptogenic focus [26]. Standardized uptake values (SUVs) for 112 target regions of interest (ROIs) were then extracted with FSL tools using a Harvard-Oxford atlas (HOA-112) [27]. SUV ratio (SUVR) values for these 112 ROIs were computed based on the normalization of SUVs to the SUV of the cerebellum as a whole.

The BRAPH toolbox was used for graph theory analyses in this study [28]. Partial correlation coefficients for SUVR values for each brain region pair were calculated in patients to generate an interregional correlation matrix (112 × 112) for each group, controlling for the impact of both age and gender. Weighted brain networks were constructed with these matrices, with nodes corresponding to regions of the brain and links between these nodes being established based on weighted correlation connections. Given that negative correlations remain subject to controversy, this study only considered positive correlations [29].

Initially, global topological indices, including average degree and strength, characteristic path length, global efficiency, local efficiency, mean clustering coefficients, modularity, assortative coefficients, and the small-worldness indices, were compared among groups [30,31], after which betweenness centrality was examined to assess local network topology [32,33]. 

### 2.4. Statistical Analysis

Quantitative data that were or were not normally distributed (Lilliefors test) were compared using Student’s *t*-tests or non-parametric Mann-Whitney *U* tests, whereas categorical data were compared with Fisher’s exact test or chi-square tests. These two respective data types were reported as means ± standard deviation (SD) and frequencies with percentages. SUVR values for each ROI were then extracted and compared among groups using one-way analysis of variance (ANOVA) corrected by Bonferroni’s multiple comparison post hoc analysis. Permutation tests with 3000 permutations were employed when comparing network measurements. *p*-values were calculated based on the fraction of permuted differences exceeding the actual difference. A two-sided *p* < 0.05 was the significance threshold. In the analysis of local network topology, an FDR correction for multiple comparisons (FDR < 0.05) was applied. The MATLAB Statistical Box (MATLAB R2021a, MathWorks) was used for all statistical comparisons.

## 3. Results

### 3.1. Demographic Data

Participant demographic and clinical characteristics are summarized in Table 1. In total, this study enrolled 54 patients with unilateral MTLE (25 males and 29 females), including 27 each with and without FBTCS. Of the FBTCS+ patients (12 males and 15 females), the respective ages at seizure onset and epilepsy duration were 10.8 ± 5.9 years and 14.3 ± 6.8 years, respectively. These patients included 15 and 12 with left and right MTLE, respectively. Of the FBTCS− patients (13 males and 14 females), the respective ages at seizure onset and duration of epilepsy were 13.8 ± 5.0 years and 14.0 ± 7.4 years, respectively. These patients included 12 and 15 with left and right MTLE, respectively. In the FBTCS+ group, 24 patients (88.9%) achieved seizure freedom with a mean follow-up of 4.8 years. For FBTCS− patients, 23 of the 27 patients (85.2%) reported being seizure-free (mean follow-up period after surgery: 4.7 years). No differences in clinical or demographic variables, including age, gender, age at seizure onset, duration of epilepsy, handedness, MTLE sidedness, and seizure outcomes, were observed when comparing FBTCS− and FBTCS+ patients (all *p* > 0.05, Table 1). There were also no differences in age or gender when comparing MTLE patients to the healthy control group.

### 3.2. Differences in SUVR Values

Relative to healthy controls, those in the FBTCS+ and FBTCS− groups exhibited a range of altered SUVR values throughout the brain. Specifically, 43 and 49 brain regions in the FBTCS+ and FBTCS− groups had significantly reduced SUVR values relative to the healthy control group (Supplementary Table S1). 38 brain regions in both groups showed a significant difference, while 5 and 11, respectively, only differed in FBTCS+ and FBTCS− patients. Ipsilateral 6 brain regions exhibited a significantly lower SUVR in FBTCS+ patients relative to FBTCS− patients, including the anterior division of the 3 temporal gyri, the posterior division of the middle and inferior temporal gyri, and the temporooccipital part of the middle temporal gyrus (Supplementary Table S1).

### 3.3. Brain Metabolic Network Abnormalities in MTLE Patients with FBTCS Relative to Healthy Controls

As compared to the healthy control group, FBTCS+ patients exhibited significant network topology changes at both the local and global levels. While a lower assortative coefficient was evident in the FBTCS+ group relative to the control group (−0.045 vs. −0.013, *p* = 0.031), no other global network measurements were altered (Table 2). 

**Figure 2:**
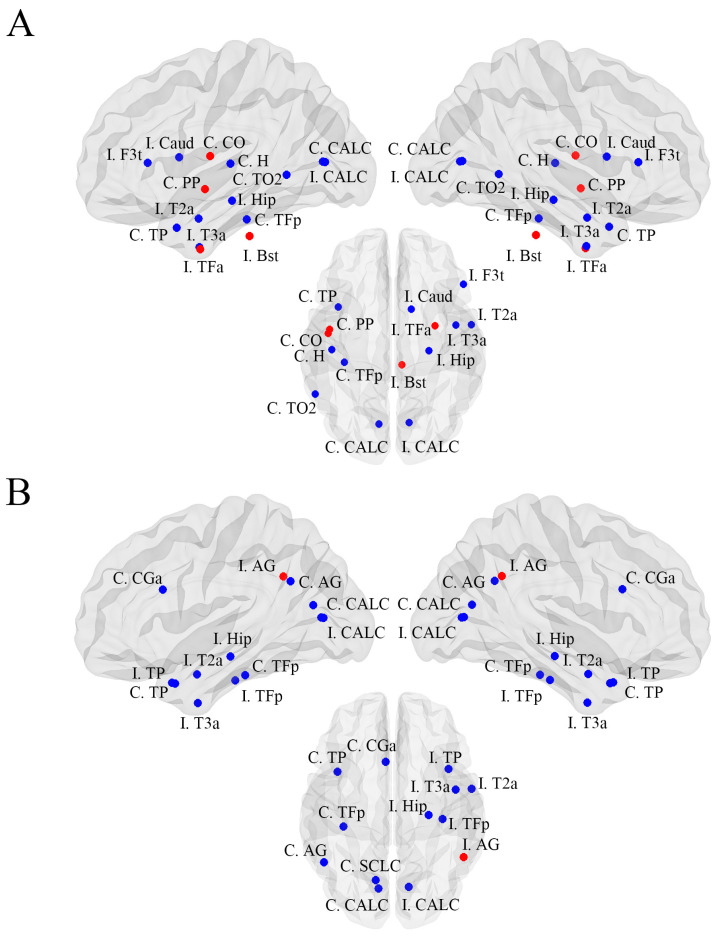
Alterations in local metabolic connectivity between FBTCS+ patients (**A**) and FBTCS− patients (**B**) relative to healthy control subjects. Red and blue circles, respectively, indicate nodes with increased and decreased betweenness centrality when comparing MTLE patients to healthy controls. TP: temporal pole; F3t: inferior frontal gyrus, pars triangularis; T2a: middle temporal gyrus, anterior division; TO2: middle temporal gyrus, temporooccipital part; T3a: middle temporal gyrus, anterior division; AG: angular gyrus; CALC: intracalcarine cortex; CGa: cingulate gyrus, anterior division; TFa: temporal fusiform cortex, anterior division; TFp: temporal fusiform cortex, posterior division; CO: central opercular cortex; PP: planum polare; H: Heschls gyrus (includes H1 and H2); SCLC: supracalcarine cortex; Bst: brain-stem; Caud: caudate; Hip: hippocampus; C: contralateral; I: ipsilateral.

With respect to local topological features, 15 brain regions exhibited significantly altered betweenness centrality in FBTCS+ patients (Figure 2A) (Table 3). Specifically, the betweenness centrality of the ipsilateral temporal fusiform cortex, contralateral central opercular cortex, contralateral planum polare, and ipsilateral brain stem was increased, whereas that of the ipsilateral inferior frontal gyrus, contralateral temporal pole, ipsilateral middle temporal gyrus, contralateral middle temporal gyrus, ipsilateral inferior temporal gyrus, bilateral intracalcarine cortex, contralateral temporal fusiform cortex, contralateral Heschel’s gyrus, ipsilateral caudate, and ipsilateral hippocampus was reduced.

### 3.4. Brain Metabolic Network Abnormalities in FBTCS− Patients Relative to Healthy Controls

No differences in global network measurements were evident when comparing FBTCS− patients to the healthy control group. Significant changes in betweenness centrality were observed in 13 regions of the brain when comparing these two groups (Figure 2B) (Table 3). A reduction in betweenness centrality was evident in 12 regions, including the bilateral temporal pole, ipsilateral middle temporal gyrus, ipsilateral inferior temporal cortex, contralateral angular gyrus, bilateral intracalcarine cortex, contralateral anterior cingulate gyrus, bilateral fusiform cortex, contralateral supracalcarine cortex, and ipsilateral hippocampus, whereas the betweenness centrality in the ipsilateral angular gyrus was increased.

### 3.5. Differences in Brain Metabolic Networks between Patients with and without FBTCS

**Figure 3:**
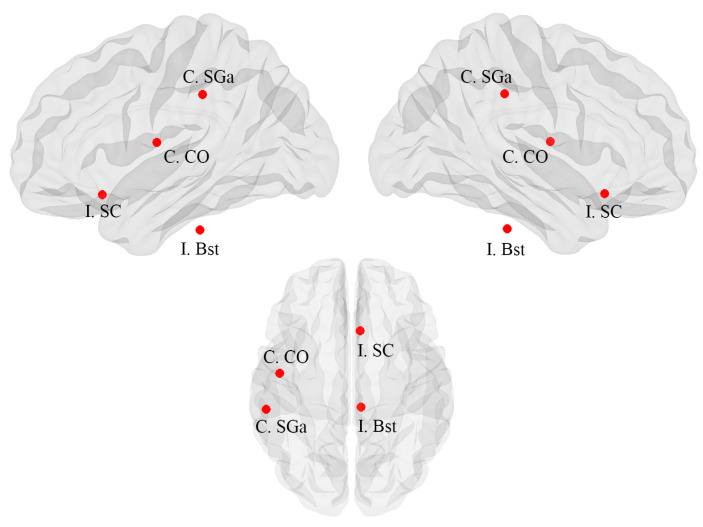
Changes in local metabolic connectivity between FBTCS+ and FBTCS− patients. Red circles indicate nodes with increased betweenness centrality when comparing MTLE patients with FBTCS to those without FBTCS. SGa: supramarginal gyrus, anterior division; CO: central opercular cortex; SC: subcallosal cortex; Bst: brain-stem; C: contralateral; I: ipsilateral.

Only local changes in network topology were significant when comparing MTLE patients with and without FBTCS. Specifically, FBTCS+ patients exhibited significant increases in betweenness centrality in the ipsilateral supramarginal gyrus, contralateral central opercular cortex, ipsilateral subcallosal cortex, and ipsilateral brain stem (Figure 3; Table 3).

## 4. Discussion

In this study, metabolic network alterations were explored in MTLE patients through FDG-PET analyses. Comparisons of healthy control subjects with MTLE patients with FBTCS revealed significant topological changes in the brain networks at the local and global levels. In contrast, only local network topology alterations were detected in FBTCS− patients relative to healthy controls. FBTCS+ and FBTCS− patients also exhibited certain local topological changes in brain networks relative to one another.

### 4.1. Brain Metabolism in Patients with MTLE

PET scans are important tools for the presurgical assessment of epilepsy patients, offering an effective means of epileptogenic temporal lobe localization in ~80% of TLE patients [34]. However, hypometabolic abnormalities evident during PET scanning may exceed those structural abnormalities observed via MRI and may not be confined to the temporal lobe, also affecting local or distant subcortical and cortical areas in some cases. Shim et al. evaluated TLE patients with and without HS and found that the extent of hypometabolism was more extensive in those patients with HS relative to healthy control subjects [35]. Those TLE patients without HS, in contrast, presented with hypometabolism in a smaller range of brain regions. Wong et al. found that MTLE patients commonly exhibit extratemporal hypometabolism, with the most frequently involved areas including the ipsilateral insular and frontal lobes [36]. They further found that secondarily generalized tonic-clonic seizures (SGTCS) were related to more extensive remote hypometabolism. In the present study, hypometabolic regions were found to exhibit a similar extent when comparing FBTCS+ and FBTCS− patients. This may be attributable to the multifactorial nature of hypometabolism, including the fact that such remote hypometabolism is related to poorer surgical outcomes in individuals with MTLE [36].

Secondary generalized seizures have been found to not involve all regions of the brain in some studies. Rektor et al. found the lateral temporal neocortex to exhibit the most prominent intracranial rating scale results in the context of ictal EEG analyses of secondary generalization, with varying degrees of changes in other cortical regions [37]. Yoo et al. further confirmed more frequent lateral temporal region involvement as compared to other areas of the cortex [38]. Neocortical TLE cases are more likely to exhibit SGTCS evolution relative to MTLE cases [39]. In this study, FBTCS+ patients were found to exhibit a lower SUVR value in the ipsilateral 6 brain regions in the temporal neocortex as compared to FBTCS− patients, suggesting a potential functional role for the temporal neocortex as a node involved in ictal spreading to other areas of the brain, thereby contributing to secondary generalization [38].

The thalamus has been established as an important regulator of primary and secondary GTCS, serving to help synchronize abnormal cortico-subcortical discharges, generate tonic-clonic motor activity, and contribute to epilepsy-associated impairment of consciousness. Thalamic structural anomalies have been detected in idiopathic generalized epilepsy patients [6]. Yang et al. also further observed a higher degree of medial thalamic atrophy among MTLE patients with SGTCS as compared to SGTCS- patients. Moreover, some PET-based studies have examined thalamic hypometabolism in TLE patients, with early reports employing quantitative or visual analytical techniques reporting hypometabolism in the thalamus ipsilateral to seizure sidedness in TLE patients [36,40]. In line with work performed previously by Van Bogaert et al. [41], hypometabolism was also observed in the subcortical structures, including the thalamus, caudate nucleus, and putamen, of MTLE patients with and without FBTCS. This suggests that while there is some variation in the degree of network involvement in this patient population, thalamic involvement is generally evident.

### 4.2. Brain Metabolic Network Alterations

Efforts to assess metabolic connectivity are focused on using PET data to identify functional interactions between brain regions. In contrast to fMRI-based approaches, this strategy exhibits less dependence on neurovascular coupling and provides insight into stable neuronal activity during the time over which recordings are made [42]. The popularity of these FDG-PET-based metabolic connectivity analyses has grown in the fields of both basic and clinical neuroscience in recent years, with several metabolic connectivity-focused models having been shown to be applicable in neurological diseases. For example, seed correlation or voxel-wise interregional correlation analysis, principal component analysis, independent component analysis, sparse inverse covariance estimation, and graph theory all exhibit advantages in this analytical setting [20].

In this study, MTLE patients with FBTCS presented with a lower assortative coefficient than healthy controls. The human brain network is extraordinarily complex, and research focused on both the dynamic and static properties of this network has advanced rapidly in recent years [31]. Graph theoretical analyses have provided a robust mathematical framework for quantifying and analyzing these brain networks, reducing network complexity to a set of parameters corresponding to facets of network topology such that both individual nodes and entire networks can be evaluated effectively. This analytical strategy has been particularly influential in the epilepsy research field, fueling important advances in the current understanding of brain network characteristics [21,30].

The present study indicated a significantly lower assortative coefficient in FBTCS+ patients when compared to healthy controls. While this coefficient did not differ significantly between MTLE patients with and without FBTCS, a trend towards a low value was also evident among FBTCS+ patients. This assortative coefficient is used to characterize network assortativity, which refers to the tendency of nodes exhibiting similar characteristics to connect with one another [28,43]. Assortative networks are those in which nodes with similar degree values tend to be connected, whereas in disassortative networks, the connected nodes tend to exhibit different degree values. In line with the results from healthy control subjects in this study, the human brain has been reported to be disassortative [43]. While assortative networks exhibit superior percolation properties and are more robust against the removal of the node with the highest degree value, disassortative networks exhibit poorer percolation properties and are more susceptible to targeted attacks [44]. Disassortative networks may thus cause epileptogenic lesions to interfere with brain networks more readily in patients with FBTCS, whereas patients unaffected by FBTCS may exhibit networks that are better able to tolerate these epileptogenic lesions. Disassortative networks have also been shown to synchronize more effectively as compared to assortative networks [45]. Patients affected by FBTCS may thus exhibit a higher degree of brain network synchronization, contributing to SGTCS incidence. 

Betweenness centrality, which is a graph theoretical index used to gauge the impact of a given node on information flow among node pairs within a given network, was employed when assessing local metabolic networks in this study. These analyses were performed based on the presumption that information generally travels along the shortest possible path among nodes. Those nodes with high levels of betweenness centrality are thus those present along the shortest pathway connecting many sets of nodes within the established network, such that they can have broad-ranging effects on network efficiency and communication. The local metabolic networks of healthy controls and MTLE patients with and without FBTCS differed significantly, with the centrality of particular regions being increased or decreased, consistent with widespread metabolic disruptions. In the local network topology analysis, the subcortical structures were also involved. However, no significant results were observed in our analysis of the subcortical structures. Despite our efforts to explore the role of the thalamus in secondary generalization, the lack of significant findings suggests that it may not play an important role in the alterations of metabolic networks in the specific context of our study. Just one node in FBTCS− patients exhibited an increase in betweenness centrality, as compared to four in the FBTCS+ group. Four regions also exhibited an increase in betweenness centrality in FBTCS+ patients as compared to FBTCS− individuals, including the ipsilateral supramarginal gyrus, contralateral central opercular cortex, ipsilateral subcallosal cortex, and ipsilateral brain stem, suggesting that these regions may be important in the context of FBTCS genesis. Indeed, some of these regions have previously been documented as important mediators of FBTCS [24,38,46]. The SUVR values of these three regions did not differ significantly between the FBTCS+ and FBTCS− patient groups, suggesting that altered glucose uptake is not directly associated with localized metabolic network alterations.

### 4.3. Limitations

This study is the first to date to have identified differences in the brain metabolic networks of MTLE patients with and without FBTCS based upon ^18^F-FDG-PET data. Even so, these analyses are subject to certain limitations. For one, the participant sample size was somewhat small, potentially constraining the statistical power of these analyses even though significant changes in metabolic networks were ultimately identified. In addition, we integrated data from the left and right mTLE patients. We were not able to differentiate between the left and right mTLE due to the small sample size. Thirdly, the cross-sectional, single-center nature of this analysis highlights the need for future multicenter, large-scale prospective validation. Finally, while these PET covariance analyses did yield valuable insights, they relied on successfully establishing a homogenous participant group. Relatively low spatial resolution also limits these PET data owing to partial volume effects.

## 5. Conclusions

In conclusion, the results of the present study indicated that FBTCS+ patients present with a higher degree of metabolic network alterations relative to FBTCS− patients, as measured via FDG-PET analyses. These findings suggest the potential existence of epileptic networks in patients with MTLE that differ as a function of FBTCS status, thereby providing insight into the etiological basis of FBTCS.

## Figures and Tables

**Table 1:** Demographic and clinical data of participants.

	FBTCS+ Patients	Healthy Controls	Statistic	*p*-Value
Age, years	25.1 ± 5.8	27.5 ± 8.8	Independence Student’s t = −1.211	0.23
Sex, male/female	12/15	15/15	χ2 = 0.176	0.67
Age at seizure onset, years	10.8 ± 5.9	N/A	N/A	N/A
Epilepsy duration, years	14.3 ± 6.8	N/A	N/A	N/A
Handedness, left/right	4/23	4/26	χ2 = 0.026	0.83
Side, left/right	15/12	N/A	N/A	N/A
	**FBTCS** **−** **Patients**	**Healthy Controls**	**Statistic**	***p*** **-Value**
Age, years	27.8 ± 5.7	27.5 ± 8.8	Independence Student’s t = 0.139	0.89
Sex, male/female	13/14	15/15	χ2 = 0.020	0.89
Age at seizure onset, years	13.8 ± 5.0	N/A	N/A	N/A
Epilepsy duration, years	14.0 ± 7.4	N/A	N/A	N/A
Handedness, left/right	3/24	4/26	χ2 = 0.065	0.88
Side, left/right	12/15	N/A	N/A	N/A
	**FBTCS+ Patients**	**FBTCS** **−** **Patients**	**Statistic**	***p*** **-Value**
Age, years	25.1 ± 5.8	27.8 ± 5.7	Independence Student’s t = −1.721	0.09
Sex, male/female	12/15	13/14	χ2 = 0.075	0.78
Age at seizure onset, years	10.8 ± 5.9	13.8 ± 5.0	Independence Student’s t = −1.987	0.05
Epilepsy duration, years	14.3 ± 6.8	14.0 ± 7.4	Independence Student’s t = 0.135	0.89
Handedness, left/right	4/23	3/24	χ2 = 0.164	>0.99
Side, left/right	15/12	12/15	χ2 = 0.667	0.41
Follow-up, years	4.8 ± 1.5	4.7 ± 1.3	Independence Student’s t = 0.070	0.95
Engel class, I/II-IV	24/3	23/4	χ2 = 0.164	>0.99

FBTCS, focal to bilateral tonic-clonic seizures; N/A, not applicable.

**Table 2:** Global metabolic network alterations in MTLE patients with and without FBTCS as compared to healthy controls.

	FBTCS+	Healthy Controls	Difference	CI Lower	CI Upper	*p*-Value
Average degree	110.054	106.875	−3.178	−5.106	6.778	0.276
Average strength	62.530	48.726	−13.803	−18.803	18.363	0.205
Characteristic path length	1.908	2.408	0.500	−0.648	0.676	0.220
Global efficiency	0.578	0.469	−0.109	−0.130	0.133	0.179
Local efficiency	3.602	2.376	−1.223	−1.463	1.484	0.184
Mean clustering coefficient	0.544	0.422	−0.122	−0.173	0.172	0.291
Modularity	0.053	0.058	0.004	−0.046	0.040	0.828
Assortative coefficient	−0.045	−0.013	0.032	−0.031	0.027	0.031 *
Small-worldness index	0.954	0.930	−0.024	−0.071	0.079	0.583
	**FBTCS** **−**	**Healthy Controls**	**Difference**	**CI Lower**	**CI Upper**	***p*** **-Value**
Average degree	110.929	106.875	−4.054	−3.145	4.030	0.063
Average strength	68.707	48.726	−19.981	−18.275	19.237	0.081
Characteristic path length	1.733	2.408	0.675	−0.599	0.569	0.057
Global efficiency	0.625	0.469	−0.156	−0.140	0.129	0.058
Local efficiency	4.191	2.376	−1.815	−1.693	1.729	0.073
Mean clustering coefficient	0.605	0.422	−0.183	−0.168	0.175	0.079
Modularity	0.035	0.058	0.023	−0.054	0.053	0.462
Assortative coefficient	−0.029	−0.013	0.016	−0.027	0.028	0.313
Small-worldness index	0.974	0.930	−0.044	−0.054	0.059	0.170
	**FBTCS+**	**FBTCS** **−**	**Difference**	**CI Lower**	**CI Upper**	***p*** **-Value**
Average degree	110.054	110.929	0.875	−3.776	3.562	0.601
Average strength	62.530	68.707	6.177	−19.774	20.686	0.614
Characteristic path length	1.908	1.733	−0.175	−0.613	0.592	0.632
Global efficiency	0.578	0.625	0.047	−0.153	0.148	0.640
Local efficiency	3.602	4.191	0.589	−1.741	1.807	0.637
Mean clustering coefficient	0.578	0.625	0.047	−0.153	0.148	0.640
Modularity	0.054	0.035	−0.019	−0.043	0.040	0.521
Assortative coefficient	−0.045	−0.029	0.016	−0.041	0.030	0.437
Small-worldness index	0.954	0.974	0.021	−0.062	0.066	0.630

MTLE, mesial temporal lobe epilepsy; FBTCS, focal to bilateral tonic-clonic seizures; C: contralateral; I: ipsilateral. * *p* < 0.05 indicates a significant difference.

**Table 3:** Significant local metabolic network alterations in MTLE patients with and without FBTCS as compared to healthy controls.

	FBTCS+	Healthy Controls	Difference	CI Lower	CI Upper	*p*-Value
I. Inferior Frontal Gyrus, pars triangularis	0.000	0.012	0.012	−0.008	0.007	0.027
C. Temporal Pole	0.000	0.019	0.019	−0.008	0.007	0.003
I. Middle Temporal Gyrus, anterior division	0.000	0.002	0.001	0.000	0.000	0.005
C. Middle Temporal Gyrus, temporooccipital part	0.000	0.008	0.008	−0.006	0.005	0.038
I. Inferior Temporal Gyrus, anterior division	0.000	0.002	0.002	−0.001	0.001	0.021
C. Intracalcarine Cortex	0.000	0.011	0.011	−0.001	0.001	0.001
I. Intracalcarine Cortex	0.000	0.003	0.003	−0.002	0.002	0.032
I. Temporal Fusiform Cortex, anterior division	0.002	0.000	−0.002	−0.002	0.002	0.028
C. Temporal Fusiform Cortex, posterior division	0.000	0.021	0.021	−0.012	0.010	0.013
C. Central Opercular Cortex	0.005	0.000	−0.005	−0.003	0.004	0.036
C. Planum Polare	0.007	0.000	−0.007	−0.003	0.002	0.013
C. Heschls Gyrus	0.000	0.011	0.011	−0.008	0.007	0.011
I. Brain-Stem	0.003	0.000	−0.003	−0.002	0.001	0.032
I. Caudate	0.000	0.002	0.002	−0.001	0.000	0.035
I. Hippocampus	0.000	0.026	0.026	−0.013	0.012	0.001
	**FBTCS** **−**	**Healthy Controls**	**Difference**	**CI Lower**	**CI Upper**	***p*** **-Value**
C. Temporal Pole	0.000	0.019	0.019	−0.010	0.009	0.003
I. Temporal Pole	0.000	0.007	0.007	0.000	0.000	0.001
I. Middle Temporal Gyrus, anterior division	0.000	0.002	0.001	0.000	0.000	0.021
I. Inferior Temporal Gyrus, anterior division	0.000	0.002	0.002	0.000	0.000	0.010
C. Angular Gyrus	0.000	0.004	0.004	−0.003	0.002	0.030
I. Angular Gyrus	0.011	0.000	−0.010	−0.006	0.005	0.024
C. Intracalcarine Cortex	0.000	0.011	0.011	−0.002	0.000	0.001
I. Intracalcarine Cortex	0.000	0.003	0.003	0.000	0.000	0.003
C. Cingulate Gyrus, anterior division	0.000	0.018	0.018	−0.010	0.008	0.016
C. Temporal Fusiform Cortex, posterior division	0.000	0.021	0.021	−0.013	0.014	0.022
I. Temporal Fusiform Cortex, posterior division	0.000	0.004	0.004	−0.003	0.003	0.042
C. Supracalcarine Cortex	0.000	0.001	0.001	0.000	0.000	0.017
Rt. Hippocampus	0.000	0.026	0.026	−0.009	0.007	0.001
	**FBTCS+**	**FBTCS−**	**Difference**	**CI lower**	**CI upper**	***p*-value**
C. Supramarginal Gyrus, anterior division	0.020	0.000	−0.020	−0.011	0.013	0.031
I. Subcallosal Cortex	0.002	0.000	−0.002	−0.002	0.002	0.043
C. Central Opercular Cortex	0.005	0.000	−0.005	−0.003	0.003	0.039
I. Brain-Stem	0.003	0.000	−0.003	−0.004	0.003	0.029

MTLE, mesial temporal lobe epilepsy; FBTCS, focal to bilateral tonic-clonic seizures; C: contralateral; I: ipsilateral.

## Data Availability

The data that supports the findings of this study are available from the corresponding author upon reasonable request.

## Supplementary Materials

The following supporting information can be downloaded at: https://www.mdpi.com/article/10.3390/brainsci13091239/s1. Table S1: The standardized uptake value ratio of regions of interest using a Harvard-Oxford atlas (nodes) in the mesial temporal lobe of epilepsy patients with and without FBTCS and healthy controls.
