# Supplementary material for: Altered Metabolic Networks in Mesial Temporal Lobe Epilepsy with Focal to Bilateral Seizures
Source: Brain Sci. 2023 Aug 25;13(9):1239. doi: 10.3390/brainsci13091239 (PMC10526398; doi:10.3390/brainsci13091239)
Supplement: Supplementary file 1 [file brainsci-13-01239-s001.zip › brainsci-2507643-supplementary.pdf]

Suppl. Table S1. The standardized uptake values ratio of regions of interest using a Harvard-Oxford atlas (Nodes) in the mesial temporal lobe epilepsy patients with and without FBTCS and healthy controls.

	FBTCS+ Healthy Controls			
	Mean Difference	CI lower	CI upper	p-value
I. Frontal Pole	-0.126	-0.207	-0.046	<0.001
C. Insular Cortex	-0.104	-0.168	-0.040	<0.001
I. Insular Cortex	-0.072	-0.131	-0.012	0.013
I. Middle Frontal Gyrus	-0.112	-0.214	-0.010	0.027
I. Inferior Frontal Gyrus, pars triangularis	-0.140	-0.238	-0.042	0.002
I. Temporal Pole	-0.194	-0.239	-0.149	<0.001
I. Superior Temporal Gyrus, anterior division	-0.195	-0.267	-0.124	<0.001
I. Superior Temporal Gyrus, posterior division	-0.151	-0.222	-0.080	<0.001
I. Middle Temporal Gyrus, anterior division	-0.308	-0.380	-0.237	<0.001
C. Middle Temporal Gyrus, posterior division	-0.075	-0.147	-0.003	0.039
I. Middle Temporal Gyrus, posterior division	-0.260	-0.330	-0.190	<0.001
I. Middle Temporal Gyrus, temporooccipital part	-0.142	-0.205	-0.079	<0.001
C. Inferior Temporal Gyrus, anterior division	-0.087	-0.166	-0.008	0.025
I. Inferior Temporal Gyrus, anterior division	-0.271	-0.337	-0.205	<0.001
I. Inferior Temporal Gyrus, posterior division	-0.208	-0.272	-0.144	<0.001
I. Inferior Temporal Gyrus, temporooccipital part	-0.069	-0.134	-0.003	0.037
I. Postcentral Gyrus	-0.093	-0.164	-0.023	0.005
C. Supramarginal Gyrus, posterior division	-0.103	-0.177	-0.028	0.003
I. Supramarginal Gyrus, posterior division	-0.085	-0.163	-0.007	0.029
I. Angular Gyrus	-0.161	-0.241	-0.081	<0.001
I. Paracingulate Gyrus	-0.140	-0.233	-0.046	0.001

C. Cingulate Gyrus, anterior division	-0.100	-0.180	-0.019	0.010
I. Cingulate Gyrus, anterior division	-0.128	-0.207	-0.048	<0.001
I. Cingulate Gyrus, posterior division	-0.204	-0.291	-0.116	<0.001
I. Precuneous Cortex	-0.134	-0.222	-0.045	0.001
I. Frontal Orbital Cortex	-0.116	-0.191	-0.042	<0.001
C. Parahippocampal Gyrus, anterior division	-0.116	-0.191	-0.042	<0.001
I. Parahippocampal Gyrus, anterior division	-0.088	-0.134	-0.041	<0.001
I. Temporal Fusiform Cortex, anterior division	-0.151	-0.203	-0.099	<0.001
I. Temporal Fusiform Cortex, posterior division	-0.168	-0.233	-0.104	<0.001
I. Temporal Occipital Fusiform Cortex	-0.078	-0.140	-0.015	0.009
I. Frontal Operculum Cortex	-0.135	-0.248	-0.022	0.014
I. Central Opercular Cortex	-0.106	-0.181	-0.031	0.003
I. Parietal Operculum Cortex	-0.103	-0.187	-0.018	0.011
I. Planum Polare	-0.137	-0.201	-0.073	<0.001
I. Heschls Gyrus (includes H1 and H2)	-0.144	-0.239	-0.049	0.001
I. Planum Temporale	-0.187	-0.269	-0.105	<0.001
I. Brain-Stem	-0.037	-0.073	-0.002	0.037
I. Thalamus	-0.143	-0.234	-0.052	<0.001
C. Caudate	-0.232	-0.354	-0.110	<0.001
I. Putamen	-0.143	-0.246	-0.040	0.003
I. Hippocampus	-0.095	-0.142	-0.049	<0.001
C. Accumbens	-0.192	-0.307	-0.077	<0.001

	FBTCS− Healthy Controls			
	Mean Difference	CI lower	CI upper	p-value
I. Frontal Pole	-0.133	-0.213	-0.052	<0.001
C. Insular Cortex	-0.105	-0.168	-0.041	<0.001
I. Insular Cortex	-0.080	-0.139	-0.021	0.004
C. Superior Frontal Gyrus	-0.109	-0.209	-0.009	0.028

I. Middle Frontal Gyrus	-0.116	-0.218	-0.013	0.021
I. Inferior Frontal Gyrus, pars triangularis	-0.125	-0.223	-0.027	0.008
I. Precentral Gyrus	-0.105	-0.188	-0.022	0.009
I. Temporal Pole	-0.185	-0.229	-0.140	<0.001
I. Superior Temporal Gyrus, anterior divisio n	-0.112	-0.184	-0.040	<0.001
I. Superior Temporal Gyrus, posterior divisi on	-0.080	-0.151	-0.009	0.022
I. Middle Temporal Gyrus, anterior division	-0.226	-0.297	-0.154	<0.001
I. Middle Temporal Gyrus, posterior division	-0.150	-0.220	-0.079	<0.001
I. Middle Temporal Gyrus, temporooccipital part	-0.064	-0.127	-0.001	0.049
I. Inferior Temporal Gyrus, anterior division	-0.201	-0.267	-0.135	<0.001
I. Inferior Temporal Gyrus, posterior division	-0.142	-0.206	-0.078	<0.001
C. Postcentral Gyrus	-0.102	-0.175	-0.029	0.003
I. Postcentral Gyrus	-0.116	-0.186	-0.045	<0.001
C. Superior Parietal Lobule	-0.091	-0.169	-0.013	0.016
C. Supramarginal Gyrus, posterior division	-0.076	-0.151	-0.002	0.042
I. Angular Gyrus	-0.083	-0.163	-0.003	0.039
C. Juxtapositional Lobule Cortex (formerly Supplementary Motor Cortex)	-0.144	-0.246	-0.042	0.003
I. Juxtapositional Lobule Cortex (formerly Supplementary Motor Cortex)	-0.191	-0.297	-0.085	<0.001
I. Paracingulate Gyrus	-0.171	-0.265	-0.078	<0.001
C. Cingulate Gyrus, anterior division	-0.137	-0.218	-0.057	<0.001
I. Cingulate Gyrus, anterior division	-0.172	-0.251	-0.093	<0.001
C. Cingulate Gyrus, posterior division	-0.107	-0.192	-0.021	0.009
I. Cingulate Gyrus, posterior division	-0.219	-0.307	-0.132	<0.001
C. Precuneous Cortex	-0.095	-0.189	-0.002	0.045
I. Precuneous Cortex	-0.136	-0.224	-0.047	0.001
I. Frontal Orbital Cortex	-0.128	-0.202	-0.053	<0.001

C. Parahippocampal Gyrus, anterior division	-0.067	-0.125	-0.008	0.020
I. Parahippocampal Gyrus, anterior division	-0.109	-0.155	-0.062	<0.001
C. Temporal Fusiform Cortex, anterior division	-0.063	-0.125	-0.001	0.043
I. Temporal Fusiform Cortex, anterior division	-0.147	-0.199	-0.094	<0.001
I. Temporal Fusiform Cortex, posterior division	-0.148	-0.212	-0.083	<0.001
I. Temporal Occipital Fusiform Cortex	-0.067	-0.129	-0.004	0.032
I. Central Opercular Cortex	-0.104	-0.179	-0.030	0.003
I. Parietal Operculum Cortex	-0.111	-0.195	-0.026	0.006
I. Planum Polare	-0.109	-0.173	-0.045	<0.001
I. Heschls Gyrus (includes H1 and H2)	-0.123	-0.218	-0.028	0.007
I. Planum Temporale	-0.140	-0.222	-0.058	<0.001
I. Brain-Stem	-0.041	-0.076	-0.005	0.019
I. Thalamus	-0.126	-0.217	-0.035	0.004
C. Caudate	-0.241	-0.363	-0.119	<0.001
C. Putamen	-0.154	-0.254	-0.055	<0.001
I. Putamen	-0.205	-0.308	-0.102	<0.001
C. Pallidum	-0.086	-0.166	-0.005	0.033
I. Hippocampus	-0.106	-0.152	-0.059	<0.001
C. Accumbens	-0.204	-0.319	-0.089	<0.001

	FBTCS+ FBTCS−			
	Mean Difference	CI lower	CI upper	p-value
I. Superior Temporal Gyrus, anterior division	-0.084	-0.157	-0.010	0.021
I. Middle Temporal Gyrus, anterior division	-0.083	-0.156	-0.009	0.022
I. Middle Temporal Gyrus, posterior division	-0.110	-0.183	-0.038	0.001
I. Middle Temporal Gyrus, temporooccipital part	-0.079	-0.143	-0.014	0.012
I. Inferior Temporal Gyrus, anterior division	-0.070	-0.137	-0.002	0.042
I. Inferior Temporal Gyrus, posterior division	-0.066	-0.132	-0.001	0.047

Statistical significance was evaluated by one way ANOVA with Bonferroni post hoc test for multiple co mparisons. The mean difference is significant at the 0.05 level.

FBTCS: focal to bilateral tonic-clonic seizures; CI: confidence interval; I: ipsilateral hemisphere; C: contralateral hemisphere.

*Only significant values are included in the table.
